# Efficient Inactivation of SARS-CoV-2 and Other RNA or DNA Viruses with Blue LED Light

**DOI:** 10.3390/pathogens10121590

**Published:** 2021-12-08

**Authors:** Chiara Terrosi, Gabriele Anichini, Jean Denis Docquier, Gianni Gori Savellini, Claudia Gandolfo, Francesco Saverio Pavone, Maria Grazia Cusi

**Affiliations:** 1 Department of Medical Biotechnologies, University of Siena, 53100 Siena, Italy; chiara.terrosi@unisi.it (C.T.); gabriele.anichini@student.unisi.it (G.A.); jddocquier@unisi.it (J.D.D.); gianni.gori@unisi.it (G.G.S.); claudia.gandolfo@unisi.it (C.G.); 2 Department of Physics and Astronomy, European Laboratory for Non Linear Spectroscopy (LENS), University of Florence, 50121 Florence, Italy; francesco.pavone@unifi.it

**Keywords:** blue LED light, SARS-CoV-2, adenovirus, respiratory syncytial virus, viral inactivation

## Abstract

Blue LED light has proven to have a powerful bacteria-killing ability; however, little is known about its mechanism of virucidal activity. Therefore, we analyzed the effect of blue light on different respiratory viruses, such as adenovirus, respiratory syncytial virus and SARS-CoV-2. The exposure of samples to a blue LED light with a wavelength of 420 nm (i.e., in the visible range) at 20 mW/cm^2^ of irradiance for 15 min appeared optimal and resulted in the complete inactivation of the viral load. These results were similar for all the three viruses, demonstrating that both enveloped and naked viruses could be efficiently inactivated with blue LED light, regardless of the presence of envelope and of the viral genome nature (DNA or RNA). Moreover, we provided some explanations to the mechanisms by which the blue LED light could exert its antiviral activity. The development of such safe and low-cost light-based devices appears to be of fundamental utility for limiting viral spread and for sanitizing small environments, objects and surfaces, especially in the pandemic era.

## 1. Introduction

A wide range of evidences about the use of blue light to inactivate pathogenic bacteria is currently present in literature [1,2]. In the past, several microbial species were studied for blue light antimicrobial activity in the spectral range of 400–470 nm, including Gram-positive and Gram-negative bacteria, mycobacteria and fungi. These studies were performed both in vitro and in vivo (preclinical studies and clinical trials) [3,4,5,6,7,8,9,10,11,12,13,14].

Antimicrobial properties of the blue light are the result of the absorption of these wavelengths by porphyrins and other chromophores within bacteria, such as flavins, leading to photochemical production of singlet oxygen and other reactive oxygen species (ROS). Bacterial exposure to ROS results in non-specific oxidative damage to vital structures and causes microbial inactivation [7,8,9,10,11,14,15,16,17,18].

Whilst the blue light effectiveness for bacterial inactivation is well studied in literature, there is a limited number of studies regarding possible virucidal effects of these wavelengths. A study on the inactivation efficacy of blue light (405 nm) on the bacteriophage/C31 indicated that the phage was susceptible to high doses of 405 nm light [19]. Richardson et al. showed that light, particularly the blue-violet part of the visible spectrum (wavelength 420–430 nm), is responsible for murine leukemia virus inactivation [20]. Likewise, another study demonstrated that a high dose of 405 nm light had a virucidal effect on feline calicivirus [21]. It is of paramount importance to find an effective tool with virucidal activity, to limit the virus spreading by aerosol and its survival on surfaces. However, as the mechanism of the inactivation through light is not elucidated, current knowledge on the antiviral efficacy of blue light requires further investigation. In this study, we analyzed the effect of blue light on some viruses, such as adenovirus, respiratory syncytial virus and SARS-CoV-2. They are respiratory viruses that can be transmitted via aerosol [22,23,24]. SARS CoV-2, in particular, can survive in the environment and on surfaces for long time [24]. Blue light showed virucidal activity on all tested viruses. 

## 2. Results and Discussion

Inactivation of microorganisms, and in particular of viruses that can be transmitted via aerosol, represents a crucial aspect of infection prevention and control procedures. In this study, we analyzed the virucidal activity of blue LED light against enveloped single strand RNA viruses, such as the respiratory syncytial virus and SARS-CoV-2, and a naked double strand DNA virus, such as the human adenovirus. We decided to test both enveloped and naked viruses in order to understand whether the envelope could be targeted with blue light leading to virus inactivation. 

**Figure 1:**
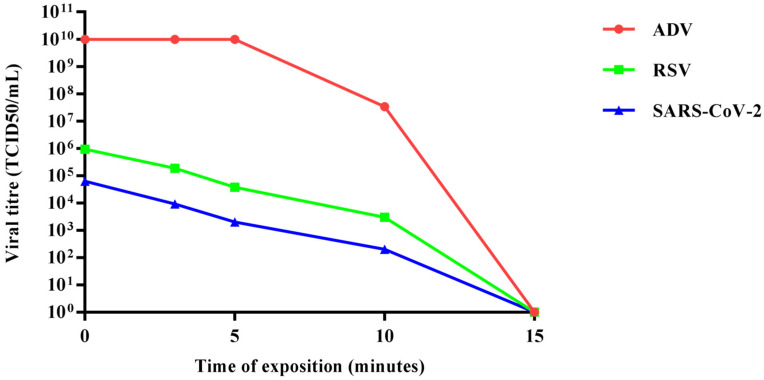
Representation of viral killing over time of adenovirus, respiratory syncytial virus and SARS-CoV-2 irradiated by blue LED light.

Preliminary trials were carried out to identify the optimal conditions (wavelength, exposure and power) for virus inactivation. The exposure of samples to a blue LED light with a wavelength of 420 nm (i.e., in the visible range) at 120 mW/cm^2^ of irradiance for 15 min appeared optimal and resulted in the complete inactivation of the viral load (measured as described in the Materials and Methods section) for all viruses treated in this study (Figure 1). 

**Figure 2:**
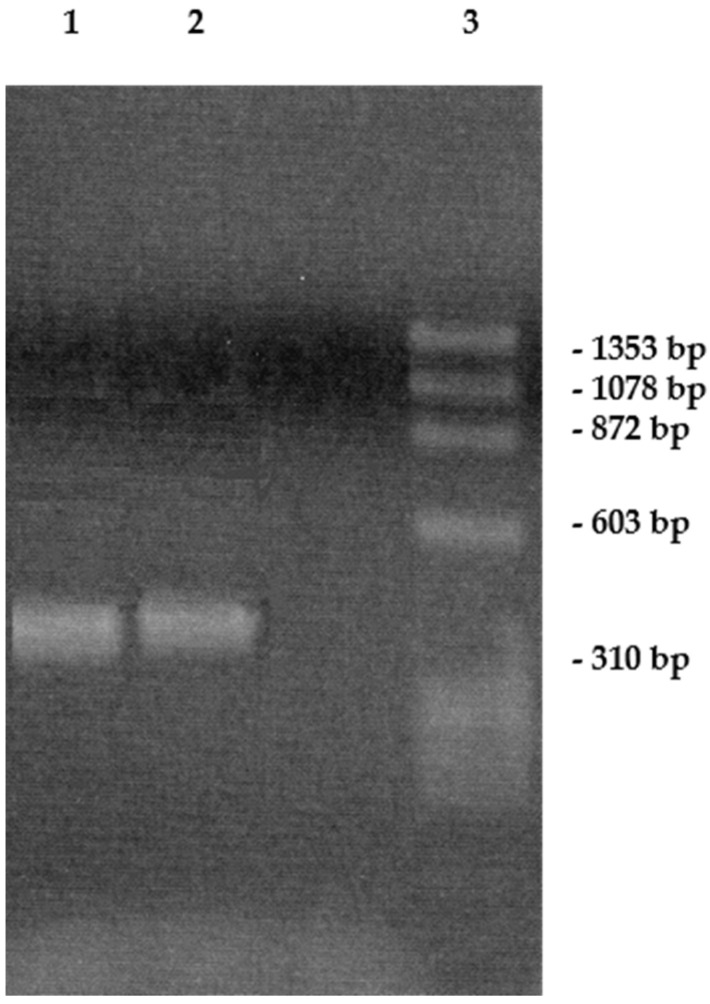
Agarose gel electrophoresis of irradiated SARS-CoV-2 (1), and non-irradiated SARS-CoV-2 (2). Standard is Thermo Scientific phiX174 DNA/BsuRI (HaeIII) Marker 9 (3).

The results demonstrated that both enveloped and naked viruses could be efficiently inactivated by blue LED light, regardless of the presence of envelope or of the viral genome nature (DNA or RNA). In order to understand whether blue LED light could affect the integrity of the viral genome after LED irradiation, SARS-CoV-2 RNA was extracted and subjected to RT-PCR analysis. An amplified product of the expected size (≈500 bps) was obtained as well as for the non-irradiated virus (Figure 2), indicating that the viral nucleic acid was likely not degraded at 120 mW for 15 min.

**Figure 3:**
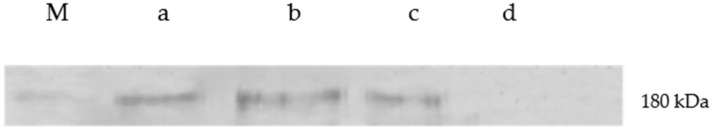
Immunoblot of transport medium + irradiated spike (**a**), irradiated transport medium + untreated spike (**b**), spike in water (**c**), irradiated transport medium (**d**), using anti-spike monoclonal antibody, as specified in Materials and Methods.

Likewise, in order to investigate whether blue light could denature the proteins, we performed an immunoblot of the recombinant spike suspended in H_2_O, after LED exposure, using an anti-spike monoclonal antibody. We did not observe a denaturing effect of blue light on the protein, which maintained the same molecular weight and showed the same profile of the control represented by the non-irradiated spike (Figure 3).

Thus, we tested the effect of LED on the nature of the matrix surrounding SARS-CoV-2. 

Since the tested viruses were all in DMEM medium, likely containing photosensitizers, such as dye (phenol red) and porphyrins, their inactivation might rely on the presence of substances, whose exposure to blue LED light would generate reactive oxygen species (ROS) and, thus, oxidation of various molecules. It is known that cell membrane is the major target of ROS upon irradiation, causing alterations of the lipid structure. The envelope, being mostly constituted of phospholipids, could be subjected to the same fate. Moreover, ROS may induce damage of the proteins leading to their denaturation, thus explaining the effect of blue light on both enveloped and naked viruses [25,26,27]. 

**Figure 4:**
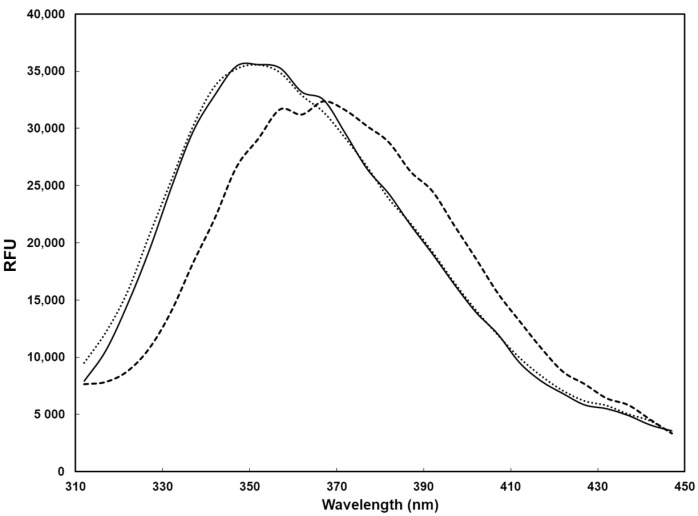
Fluorescence spectrum (excitation wavelength, 280 nm) of the recombinant trimeric SARS-CoV-2 spike protein (0.3 μM). Solid line, spectrum of the native protein in water; dashed line, spectrum of the denatured protein (obtained after incubation in the presence of 4.5 M GdmCl); dotted line, spectrum of the protein after exposure to blue LED light.

Therefore, we focused on SARS-CoV-2, in order to see whether the presence of photosensitizers was necessary to activate LED. To this aim, we collected a nasopharyngeal swab from a COVID-19 positive subject in physiological solution (NaCl 0.9%), not containing any photosensitizers. Half of the sample was left at room temperature, the other part was irradiated as described above. Surprisingly, the viral load in the irradiated sample was reduced by 3 log (TCID_50_ 2 × 10^2^/mL) with respect to the untreated virus (TCID_50_ 2 × 10^5^/mL) (*p* < 0.05). We deduced that the liquid matrix where the swab was soaked, had a role in the viral inactivation. Organic materials, such as highly glycosylated mucins, containing cysteine-rich domains, which form disulfide bonds between mucin dimers [28], and other substances present in the biological swab or human respiratory droplets could behave as photosensitizers [29]. These factors allowed the activation of LED and consequently the oxidation of molecules, damaging the virus itself. To confirm these data, we performed experiments on the SARS-CoV-2 recombinant trimeric spike, suspended in sterile water or in UTM medium and irradiated with LED blue light for 15 min. First, the fluorescence emission spectra of both the native and unfolded (after incubation of the protein sample in 4.5 M guanidinium chloride, commonly used as a chaotropic agent leading to protein denaturation) trimeric SARS-CoV-2 spike protein were obtained (Figure 4). Upon denaturation, a blue shift was clearly observed, with the maximum wavelength of emission varying from 345 to 365 nm, while the fluorescence intensity was less significantly affected. However, the fluorescence spectrum of the protein sample resuspended in water was not affected after exposure to blue LED light (Figure 4), supporting the hypothesis that irradiation with blue light would not directly induce the denaturation of the trimeric spike protein and would support the role of photosensitizers present in the liquid matrix. 

**Figure 5:**
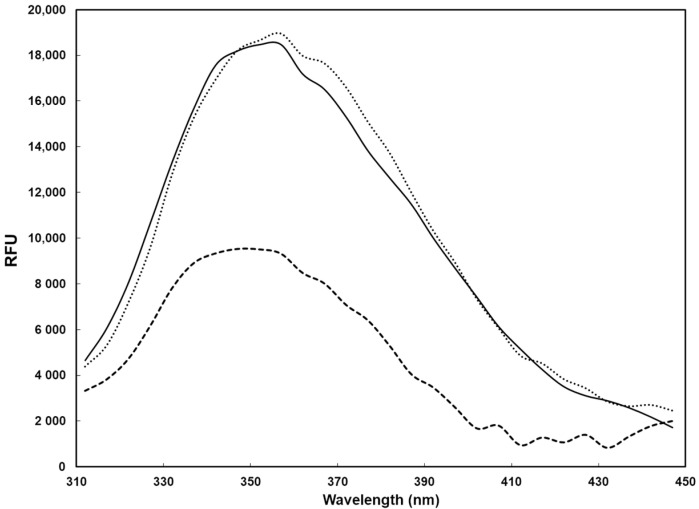
Fluorescence spectrum (excitation wavelength, 280 nm) of the recombinant trimeric SARS-CoV-2 spike protein (0.3 μM) in UTM culture medium. Solid line, spectrum of the protein in the culture medium without exposure; dashed line, spectrum of the protein in the culture medium after exposure of the sample to blue LED light; dotted line, spectrum of the protein after its addition to culture medium exposed to blue LED light.

To further probe this hypothesis, the fluorescence spectra of the trimeric spike protein suspended in transport medium (UTM) were analyzed both before and after exposure to blue LED light (Figure 5). A significant reduction of fluorescence intensity was observed upon exposure to blue LED light, indicating that the irradiation had an effect on the protein, although the blue shift characterizing the denatured spike protein was not observed in this case. In addition, the spike protein added to a culture medium, previously subjected to blue LED light irradiation, showed no visible alteration of the fluorescence spectrum. Overall, these data indicate that the alteration of the trimeric spike protein likely requires the presence of photosensitizers contained in the culture medium. Since many viruses spread via droplet transmission, containing substances that can function as photosensitizers, the use of blue LED light can be exploited for virucidal activity. In conclusion, we provided some explanations to the mechanisms by which the blue LED light exerts its antiviral activity. Moreover, the development of safe low-cost light-based devices with the capability to inactivate viruses, sanitize equipment, hospital areas, particularly in pandemic era, appears of fundamental utility to limit the spread of viruses. The relevance of the decontamination of the environment, such as patient-care rooms, using blue light, will be evaluated by measuring its efficacy to reduce transmission of infectious pathogens. 

## 3. Materials and Methods

### 3.1. Blue Light LED Instrument

The illuminator prototype used during the experiment was equipped of an array of blue LED emitting at 410–430 nm (Emoled Srl—Florence, Italy). The LED-based illuminator prototype was equipped on a steel stand in order to keep it at a distance of 4 cm from the samples during the illumination, giving an irradiance of 20 mW/cm^2^ on the sample. The samples were irradiated for 3, 5, 10 or 15 min. The temperature of the medium was monitored by Ing. Domenico Alfieri; the temperature was not higher than 30 °C for the duration of the treatment.

### 3.2. Cells and Viruses

Vero E6 cells (ATCC CRL-1586) were cultured in Dulbecco’s modified Eagle’s medium (DMEM with phenol red dye) (Lonza, Milan, Italy) supplemented with 100 U/mL penicillin/streptomycin (Hyclone Europe, Milan, Italy) and 5% heat-inactivated fetal calf serum (FCS) (Lonza), at 37 °C. SARS-CoV-2 was isolated on Vero E6 cells from a clinical specimen in the Virology laboratory of ‘S. Maria alle Scotte’ Hospital in Siena, Italy. The respiratory syncytial virus (RSV) type A (ATCC VR-26) and adenovirus (VR-1516) were used for this study. All of them were suspended in DMEM.

### 3.3. Virus Exposure to the Blue LED Light

One mL of SARS-CoV-2, AdV or RSV was distributed in a Petri dish (35 × 11 mm^2^, Thermo Fisher Scientific Inc.) and exposed for 3, 5, 10 or 15 min to the blue LED light at room temperature. The temperature was monitored during the irradiation. Likewise, for the control, 1 mL of each virus was put in a Petri dish and kept unexposed to the blue LED light, for the same time points at room temperature. After that, the viral suspension was collected and virus titration was performed. Fifty μL of the suspension was ten-fold serial diluted (10–10^–8^) and distributed (in quadruplicate) in a 96 well plate. Furthermore, 50 μL (2 × 10^5^/mL) of virus-susceptible Vero E6 cells were added in each well and incubated at 37 °C, 5% CO_2_ incubator. The plate was daily observed under an optical microscope (Olympus IX51, magnification 100×). The final virus titer was evaluated after 3 days, when a cytopathic effect was evident in cells infected with the positive untreated virus control. 

The viral titer was calculated according to the ‘Reed and Muench’ method [30]. This analysis calculates the 50% infectious dose of tissue cultures (TCID_50_/mL). The test was performed in triplicate. The experiments carried out with SARS-CoV-2 were performed in BSL-3 laboratory.

### 3.4. SARS-CoV-2 Spike Protein Exposure to the Blue LED

The SARS-CoV-2 spike protein in its trimeric form (Recombinant SARS-CoV-2 Trimeric Spike Protein; Leinco Technologies, Fenton, MO, USA) was treated with blue LED light, as the virus. Therefore, the protein (0.12 μg/mL) was resuspended in complete sterile H_2_O and irradiated for 30 min. The negative control was represented by the non-treated spike protein, at the same concentration (0.12 μg/mL) in H_2_O. Likewise, the protein was resuspended in UTM medium (Copan, Italy; containing Hank’s balanced salts bovine serum albumin, L-cysteine, gelatin, sucrose, L-glutamic acid, HEPES buffer, vancomycin, amphotericin B, colistin, phenol red) and irradiated with the blue light at the same conditions. Moreover, we irradiated only the UTM medium for 30 min, following which, the protein (0.12 μg/mL) was added. Lastly, a nasopharyngeal swab drawn from a COVID-19 infected patient (BIOBANK-MIU-2010, document approved by the Ethics Committee with amendment No. 1, on 17 February 2020) soaked in 1 mL of physiological buffer (NaCl 0.9%) was treated under blue light as above described. All the samples were then collected and tested for viral titration.

### 3.5. Immunoblot

Fifty microliters of each sample were loaded on an 8% SDS-PAGE gel, then transferred to a NitroBind nitrocellulose membrane (Santa Cruz Biotechnology, Heidelberg, Germany). Briefly, membrane blocking was accomplished with 5% non-fat dry milk, then filters were incubated o/n at 4 °C with anti-SARS-CoV-2 S monoclonal antibody (Clone LGSV201; Abnova, Taipei, Taiwan) (1:2000). After being washed with PBS 0.2% Tween-20 (PBS-T), membranes were incubated with horseradish peroxidase (HRP)-conjugated secondary antibody (Merck-Millipore) (1:5000). Immunocomplexes were detected with TMB Enhanced One Component HRP Membrane Substrate (Tebu-bio, Milan, Italy). Molecular weight markers were prestained protein SHARPMASS VI (Euroclone, Pero (MI), Italy)

### 3.6. Amplification of SARS-CoV-2 Spike Gene

Nucleic acid of the treated SARS-CoV-2 sample was extracted using the EZ1 Advanced XL system (Qiagen, Hilden, Germany), according to the manufacturer’s instructions. Reverse transcription and PCR were performed using SuperScript III One-Step RT-PCR System with Platinum Taq DNA Polymerase (Invitrogen) by one cycle of 30 min at 45 °C and 2 min at 94 °C, 40 cycles of PCR, with each cycle consisting of 15 s at 94 °C, 30 s at 58 °C and 60 s at 68 °C, followed by a final elongation step for 5 min at 68 °C. Primer sequences for RT-PCR analysis were as follows:

Spike 2 For: 5′ CCTTTTGAGAGAGATATTTCAACTG 3′ (25 nt)

Spike 2 Rev: 5′ GATCAACTTACTCCTACTTGGCG 3′ (23 nt)

The final product was a fragment of 515 bps (nt 1387-1902 GeneBank No. MZ027646.1)

### 3.7. Protein Analysis Techniques

The potential of blue LED light to affect the three-dimensional structure of SARS-CoV-2 spike protein was investigated by fluorescence spectroscopy. Furthermore, 0.3 µM of SARS-CoV-2 spike protein was diluted in MilliQ water. Protein denaturation was carried out by incubating the protein sample in the presence 4.5 M guanidinium chloride (GdmCl) for up to 1 h. Fluorescence spectra (excitation wavelength, 280 nm; emission wavelength, 310–450 nm) of the native and denatured protein were recorded using an Envision microplate reader and black SpectraPlate microplates (Perkin Elmer, Waltham, Mass.) containing a final volume of 200 µL. To assess the effect of exposure to blue LED light, the protein was diluted either in MilliQ water or in the universal transport medium (Copan Italia Sp.p.A, Brescia, Italy). Irradiation conditions were as previously mentioned (see ‘Blue light LED instrument’ and ‘SARS-CoV-2 Spike Protein exposure’). 

## Data Availability

All data supporting reported results of this work are available from the corresponding author (Cusi, M.G., mariagrazia.cusi@unisi.it), upon reasonable request.
